# Effects of Patch-Size on Populations of Intertidal Limpets, *Siphonaria* spp., in a Linear Landscape

**DOI:** 10.1371/journal.pone.0052076

**Published:** 2012-12-20

**Authors:** Victoria J. Cole, Linda G. Johnson, Christopher D. McQuaid

**Affiliations:** Coastal Research Group, Department of Zoology and Entomology, Rhodes University, Grahamstown, South Africa; University of Plymouth, United Kingdom

## Abstract

Organisms with different life-histories and abilities to disperse often utilise habitat patches in different ways. We investigated the influence of the size of patches of rock (separated by stretches of sand) on the density of pulmonate limpets (*Siphonaria* spp.) along 1500 km of the linear landscape of the South African coastline. We compared the influence of patch-size on two congeneric species with different modes of development, *S. serrata* a direct developer, and *S. concinna* a planktonic developer. We tested the spatial and temporal consistency of the effects of patch-size by sampling 7 independent regions spanning the distributional range of both species of limpets, and by sampling one region at monthly intervals for 1 year. Within each region or month, 4 small patches (<20 m in length) interspersed with the 4 large patches (>60 m in length) were sampled. Across the entire geographic range and throughout the year, there were more of both species of limpets in large patches than in small patches. In most regions, there was greater variability in large patches than small patches. Variability within patches in a single region was similar throughout the year, with greater variability of both species in large than in small patches. We found little influence of the mode of development on the response of limpets to patch-size. Our findings highlight the importance of understanding patterns of distribution of species with respect to habitat heterogeneity in linear landscapes, and contradict the idea that organism mobility at an early ontogenetic stage directly affects habitat use.

## Introduction

Habitat loss and fragmentation are generally considered to be among the major threats to biodiversity [1]. Consequently the ways in which an organism perceives and reacts to habitat patchiness are important and are believed to depend strongly on the ability of an organism to disperse [2]. Dispersal can include not only adult motility, but also the effects of life-history on the ability to disperse at different ontogenetic stages. Previous studies suggest that organisms with different dispersal abilities and life-history strategies utilise different types of habitat patches [3], [4]. For example, Meyer & Posey [4] found that two species of fish with contrasting dispersal abilities and life-histories utilised patches of marshes and shallow water flats habitats differently. Information on how organisms perceive and respond to changes in a landscape, and the ways in which behavioural and life-history traits influence responses to habitat heterogeneity is therefore needed in order to predict the effects of habitat change on populations [5], [6].

The size of patches of habitat has an important influence on many ecological patterns and processes [7], [8], . Various hypotheses have been proposed to explain how populations of organisms should respond to patch-size, leading to debates about conservation and the role of small or large reserves [10]. Following island biogeography equilibrium theory [11], larger islands are expected to have more species than smaller ones due to decreased extinction and increased immigration rates as well as the availability of more habitats [12]. Under metapopulation theory, organisms are less likely to disperse and the chances of recolonisation are greater if the quality of the patch is high [2], [13]. In terrestrial landscapes, a meta-analysis of studies investigating the role of patch-size as measured by area, densities of insects and birds were positively correlated with patch-size [14]. At smaller scales, in intertidal landscapes, the occupation of patches is also dependent on their size [7], [15]. For example, colonisation by algae into cleared patches of rocky shore has been shown to be dependent on patch size such that small patches had a greater influence of grazing limpets than large patches. Subtidally, the ability of species to colonise substrata can structure assemblages on small patches, while large patches are strongly influenced by competition rather than recruitment [16].

Most studies involve two-dimensional landscapes in which dispersers among patches can move via many different routes [17]. Some landscapes, such as rivers, coasts and mountain tops/ridges, have, however, a greater tendency to be naturally fragmented, as they are essentially linear and encompass a matrix which contains small patches of different types of habitats [18]. In rivers, for example, dispersers are restricted to linear landscapes and cannot move to distant patches without passing through a series of intervening patches [19]. Across larger scales, such as along coastlines, rocky habitats are often patches separated by a sandy matrix, within a linear landscape. Coastlines also differ from the terrestrial landscapes by experiencing oceanographic processes that influence dispersal and other ecological processes [20].

This study investigates the influence of the size of patches (i.e. patches of rock) on the density of pulmonate limpets (*Siphonaria* spp.) along 1500 km of the linear landscape of the South African coastline. Although we do not aim to unravel the mechanisms influencing the population dynamics of limpets, we separate the patterns shown on large and small patches with respect to adult and juvenile limpets in an attempt to give an insight into the importance of recruitment and post-recruitment processes. Furthermore, as the characteristics of an organism influence the way in which habitat patchiness is perceived [6], [21], we investigate the influence of the mode of development by comparing congeneric species with different modes of development. Both species have very limited dispersal as adults (previous studies have shown adult *Siphonaria* to move less than 10 cm per day, [22]), being effectively unable to move between patches of rock that are isolated from one another by stretches of sand, but at earlier stages of development they differ. Both species lay benthic egg masses. *Siphonaria serrata* is a direct developer, laying egg masses from which offspring emerge as young limpets, while *Siphonaria concinna* is a planktonic developer that lays an order of magnitude more eggs [23] that hatch as larvae that are in the water column for approximately 2–3 weeks and are capable of much wider dispersal [24]. For organisms with dispersive life-stages, such as pelagic developers, the probability of encountering a large patch is greater than reaching a small patch, and thus, unless they test and reject patches, they are more likely to colonise large patches than small patches [25]. Therefore, because encounter rates of planktonic larvae would be less frequent for small patches, we predicted that the density of pelagic-developer, *S. concinna*, would be less than in large patches. In contrast, because egg masses of the direct developer hatch as crawl-away juveniles, we predicted that this effect would be weaker for *S. serrata*. As encounter rates for pelagic larvae would be rarer and less predictable for smaller patches, we hypothesised that the variability in densities of limpets in small patches would be greater for the pelagic developer *S. concinna* than for *S. serrata*, but that this difference would be reduced on large patches where larval encounters would be more regular. Variability in both species would reflect both spatial and temporal variability in patch quality, but for *S. concinna* it would also reflect the effects of dispersal. The spatial and temporal consistency of our results was determined along the1500 km distribution of the species and within one region throughout one year.

## Methods

**Figure 1:**
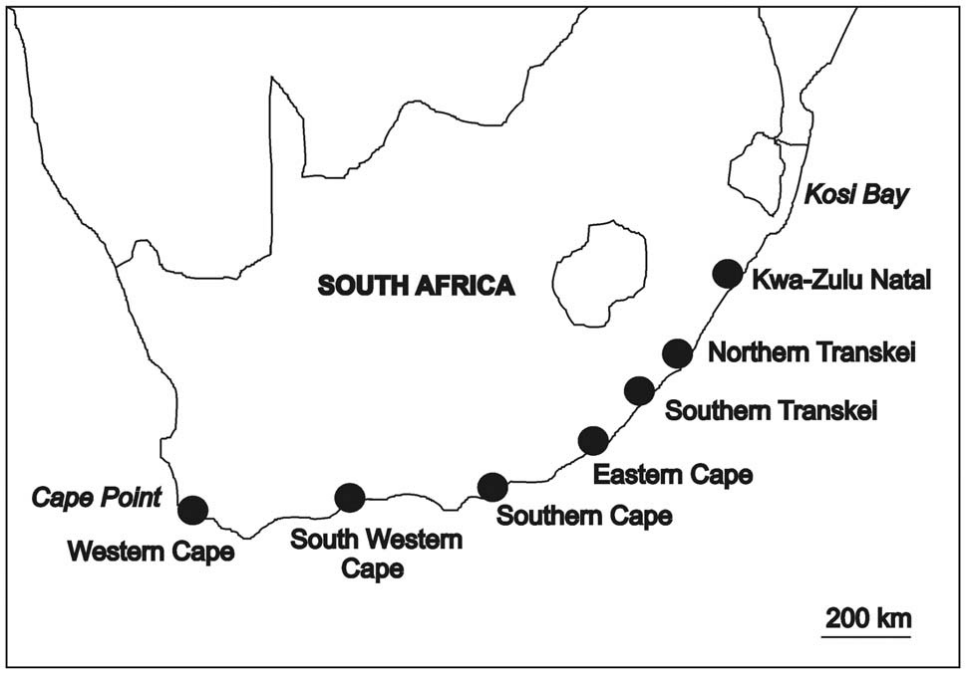
Map of South Africa showing the 7 different regions which were sampled within the geographic range of *Siphonaria concinna* and *S. serrata*. For both species, Cape Point is the western limit and Kosi Bay is the eastern limit.

*Siphonaria concinna* and *S. serrata* occur from Cape Point in the west to Kosi Bay in the east, of South Africa [24]. To test hypotheses about the effects of patch-size on densities of limpets across large spatial scales, sampling was done during spring low tides, during the Austral Spring/Summer of 2009 at seven independent regions (each regional area extended over approximately 80 km of coast) spanning a total of 1500 km of coastline. From west to east respectively these were: Western Cape, South Western Cape, Southern Cape, Eastern Cape, Southern Transkei, Northern Transkei and Kwa-Zulu Natal (Fig. 1). The type of rock differed among regions, being generally basalt or sandstone. Nevertheless, all patches were defined as being non-mobile rocks separated by at least 10 metres of sandy substratum from adjacent rocky substratum. To test hypotheses about the effects of time-courses on patch-size, the Eastern Cape patches (Fig. 1) were sampled each month for twelve months, from June 2009 till May 2010. The Eastern Cape patches were chosen as they are at the centre of distribution for both species of limpets. Although the same patches were sampled throughout the year, independent quadrats were sampled each month.

Based on a pilot study (unpublished data, L.G. Johnson 2010), small patches were defined as smaller than 20 m in length and large patches were greater than 100 m in length. Patches that occurred on the mid-shore and where both species were present were selected. The maximum intertidal length of each patch was measured. Within each region or month, 4 small patches interspersed with the 4 large patches were sampled. At each patch, for each species, six replicate 50×50 cm quadrats were haphazardly sampled on the mid-shore. Each species was sampled separately to maintain independent estimates of the two populations. Within each quadrat, all limpets were measured to the nearest half millimetre along the longest axis of the shell using vernier callipers. Juveniles were defined as limpets smaller than 15 mm and adults were larger than 15 mm [24].

Separate analyses were done for adults and juveniles as estimates of each were determined within the same quadrats. Densities were compared between small and large patches among the seven regions for *S. concinna* and *S. serrata*. The analysis was therefore a four-factor Analysis of Variance (ANOVA) with the factors: Region (random, 7 levels), Species (fixed, orthogonal, 2 levels), Size (fixed, orthogonal, 2 levels), and Patch (random, nested in Region and Size, 4 levels), and *n* = 6 replicate quadrats. Densities of *S. concinna* and *S. serrata* were compared between small and large patches at fixed monthly intervals throughout the year with a four-factor ANOVA with the factors: Month (fixed, 12 levels), Species (fixed, orthogonal, 2 levels), Size (Fixed, orthogonal, 2 levels), Patch (random, nested in Size, 4 levels), and *n* = 6 replicate quadrats. Prior to analyses data were tested for homogeneity of variances with Cochran’s test. Sqrt(x+1) transformation is appropriate for counts from quadrats [26], and for all analyses of densities when data were Sqrt(x+1) transformed, the assumption of homogeneity of variances was satisfied. As data of variances were right-skewed, Ln(x+1) transformation satisfied the assumption of homogeneity of variances. When the interaction term was non-significant (*P*>0.25), it was pooled to allow a more powerful test of individual factors [26]. *Post hoc* Student-Newman-Keuls (SNK) tests were done for significant sources of variation to examine the direction of differences relevant to hypotheses of interest.

To test for hypotheses about within patch variation, we analysed variance as the dependent factor, using similar experimental designs to those used for analyses of densities but did not include the factor “Patch”. Data consisted of 4 replicate estimates of variance from each small or large patch. All analyses were done using the Analysis of Variance programme, GMAV-5 for Windows [27].

## Results

**Figure 2:**
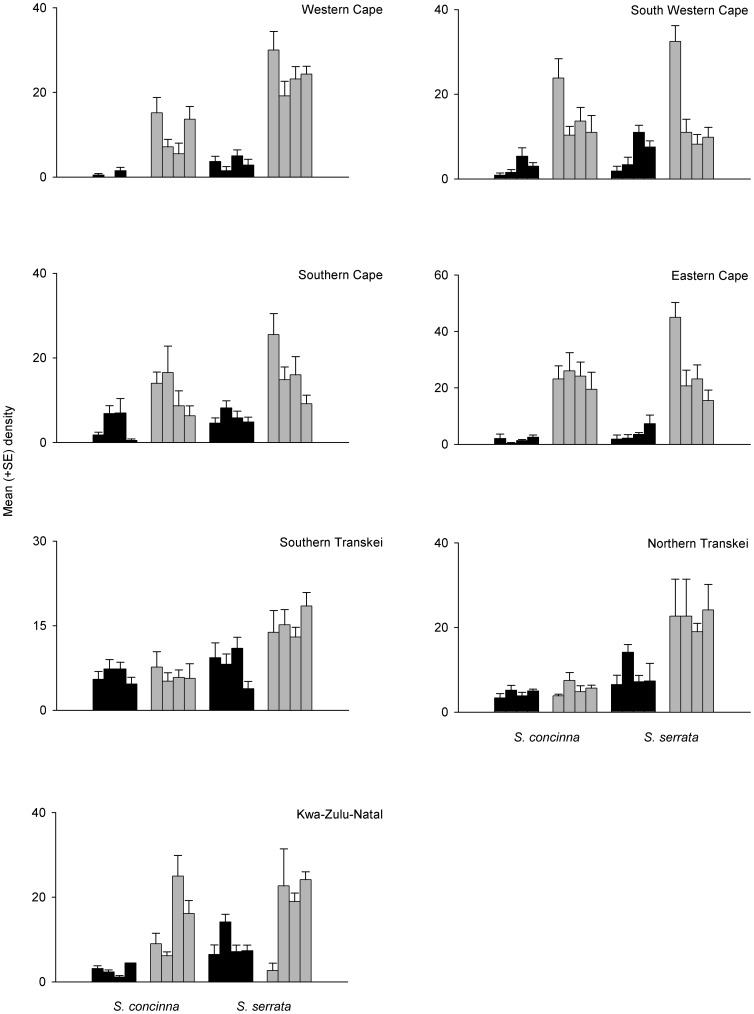
Mean (+SE; *n* = 6) density of adult *Siphonaria concinna* and *S. serrata* in small (black bars) or large patches (grey bars) in 7 different regions (Western Cape, South Western Cape, Southern Cape, Eastern Cape, Southern Transkei, Northern Transkei and Kwa-Zulu Natal).

**Figure 3:**
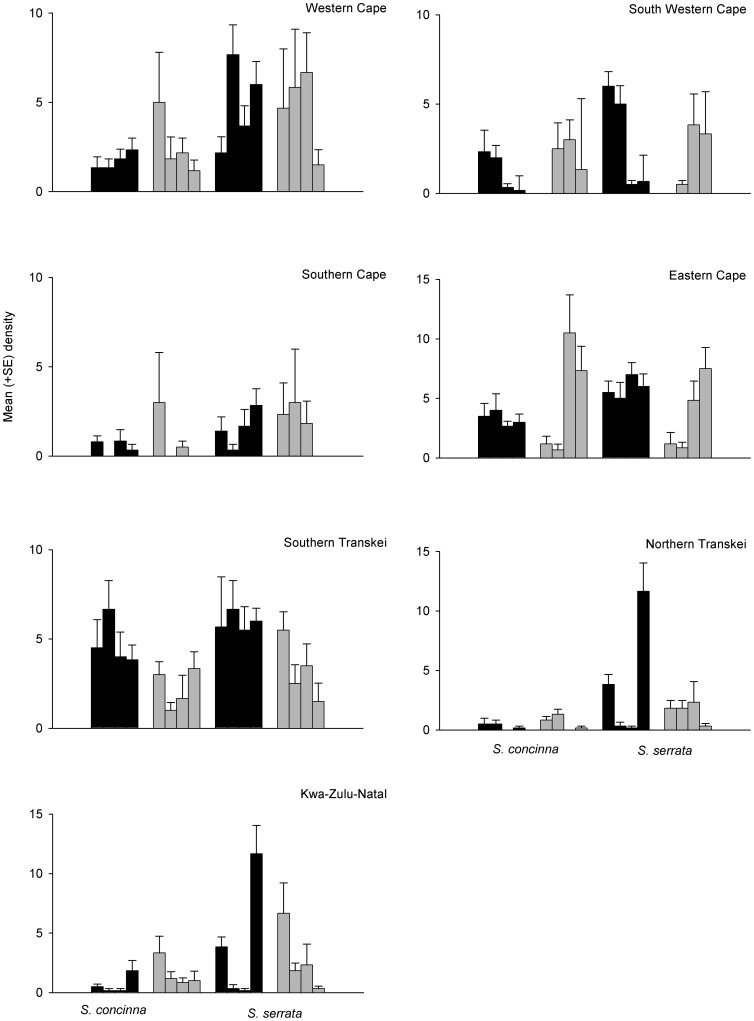
Mean (+SE; *n* = 6) density of juvenile *Siphonaria concinna* and *S. serrata* in small (black bars) or large patches (grey bars) in 7 different regions (Western Cape, South Western Cape, Southern Cape, Eastern Cape, Southern Transkei, Northern Transkei and Kwa-Zulu Natal).

When investigating differences in densities of limpets between small and large patches across their entire geographic range of South Africa, we predicted a significant interaction among the factors Species and Size. Contrary to our predictions, both species responded to small and large patches in a similar manner (Table 1, Fig. 2). There was a significant effect of Region by Size (Table 1) with a greater density of adult limpets in large patches than small patches in most regions (Fig. 2) but this was only significant in the Eastern Cape (Table 1). In Southern Transkei, there was, however, the opposite pattern, with greater densities in small patches than in large (Table 1). Clearly, there was large variability among regions and patches such that there was also a significant interaction between Region and Species and a main effect of Patch (Table 1, Fig. 2). Similarly for juvenile limpets, there was high variability among patches with a significant Species by Patch interaction and also significant interactions of Region by Species and Region by Size (Table 2, Fig. 3). Specifically, for juveniles, there were greater densities of limpets in large patches in 5 of the 7 regions (Fig. 3), although this was significant for 3 regions only (Table 2). Two regions (South Western Cape and Southern Cape) did, however, show the opposite pattern with greater densities of juveniles in small patches than in large (Table 2). Analyses of the within patch variance showed that there was a significant interaction between Region and Species and also Region and Size (Table 3). Specifically, variation was significantly greater within large patches than small patches in 4 of the 7 regions, South Western Cape, Southern Cape, Eastern Cape, and Kwa-Zulu-Natal (Table 3).

**Table 1:** Analysis of variance comparing densities of adult *Siphonaria concinna* and *S. serrata* in 7 regions along the 1500 km of the limpets’ biogeographic range in 4 small and 4 large patches.

Source	d.f.	M.S.	*F*	*P*
Region = Re	6	72.29	47.68	0.00
Species = Sp	1	25.36	10.80	0.02
Size = Si	1	7.42	0.43	0.54
Patch(Re × Si) = Pa	42	1.52	3.39	0.00
Re × Sp	6	2.35	2.77	0.02
Re × Si	6	17.21	11.35	**0.00**
Sp × Si	1	0.03	0.03	0.86
x Sp × Pa(Re × Si)	42	0.81		
x Re × Sp × Si	6	1.08		
Residual	560	0.45		
Total	671			
Pooled	48	0.85	1.90	0.00
**SNK:** Region × Size				
Western Cape, South Western Cape, Southern Cape: Small = Large				
Eastern Cape: Small<Large				
Southern Transkei: Small>Large				
Northern Transkei, Kwa-Zulu-Natal: Small = Large				

Region (Western Cape, South Western Cape, Southern Cape, Eastern Cape, Southern Transkei, Northern Transkei and Kwa-Zulu Natal) was a random factor, Species (*S. concinna* or *S. serrata*) was fixed and orthogonal, Size (small or large) was fixed and orthogonal, and Patch (4 small and 4 large) was a random factor and nested in Region and Size, and *n* = 6 replicate quadrats. Data were Sqrt(x+1) transformed to satisfy homogeneity of variances, Cochran’s Test *C* = 0.04. The Re x Sp x Si interaction term was non-significant (*P*>0.25) and was pooled with Sp x Pa(Re x Si) to allow a more powerful test of individual factors [26]. *Post hoc* Student-Newman-Keuls (SNK) tests were done for significant sources of variation to examine the direction of differences relevant to hypotheses of interest (highlighted in bold).

x Denotes *post hoc* pooling when *P*>0.25. New *F* ratios are given for those tested against the pooled terms.

**Table 2:** Analysis of variance comparing densities of juvenile *Siphonaria concinna* and *S. serrata* in 7 regions along the 1500 km of the limpets’ biogeographic range in 4 small and 4 large patches.

Source	d.f.	M.S.	*F*	*P*
Region = Re	6	4.27	0.97	0.46
Species = Sp	1	52.28	5.76	0.05
Size = Si	1	81.42	1.01	0.35
Patch(Re × Si) = Pa	42	4.41	5.35	0.00
Re × Sp	6	9.07	3.24	0.01
Re × Si	6	80.93	18.34	**0.00**
Sp × Si	1	0.25	0.04	0.84
Sp × Pa(Re × Si)	42	2.80	3.39	0.00
Re × Sp × Si	6	5.75	2.05	0.08
Residual	560	0.83		
Total	671			
**SNK**: Region × Size				
Western Cape: Small<Large				
South Western Cape: Small>Large				
Southern Cape: Small>Large				
Eastern Cape: Small<Large				
Southern Transkei: Small = Large				
Northern Transkei: Small = Large				
Kwa-Zulu-Natal: Small<Large				

Region (Western Cape, South Western Cape, Southern Cape, Eastern Cape, Southern Transkei, Northern Transkei and Kwa-Zulu Natal) was a random factor, Species (*S. concinna* or *S. serrata*) was fixed and orthogonal, Size (small or large) was fixed and orthogonal, and Patch (4 small and 4 large) was a random factor and nested in Region and Size, and *n* = 6 replicate quadrats. Data were Sqrt(x+1) transformed to satisfy homogeneity of variances, Cochran’s Test *C* = 0.05. *Post hoc* Student-Newman-Keuls (SNK) tests were done for significant sources of variation to examine the direction of differences relevant to hypotheses of interest (highlighted in bold).

**Table 3:** Analysis of variance comparing within patch variance of *Siphonaria concinna* and *S. serrata* in 7 regions along the 1500 km of the limpets’ biogeographic range in small and large patches.

Source	d.f.	M.S.	*F*	*P*
Region = Re	6	11.74	16.35	0.00
Species = Sp	1	0.44	0.12	0.74
Size = Si	1	31.66	4.01	0.09
Re × Sp	6	3.70	5.16	0.00
Re × Si	6	7.90	11.01	**0.00**
Sp × Si	1	0.12	0.17	0.68
x Re × Sp × Si	6	0.90		
x Residual	84	0.71		
Total	111			
Pooled	90	0.72		
**SNK:** Region × Size				
Western Cape: Small>Large				
South Western Cape: Small<Large				
Southern Cape: Small<Large				
Eastern Cape: Small<Large				
Southern Transkei: Small = Large				
Northern Transkei: Small = Large				
Kwa-Zulu-Natal: Small<Large				

Region (Western Cape, South Western Cape, Southern Cape, Eastern Cape, Southern Transkei, Northern Transkei and Kwa-Zulu Natal) was a random factor, Species (*S. concinna* or *S. serrata*) was fixed and orthogonal, Size (small or large) was fixed and orthogonal, and *n* = 4 replicate patches. Data were Ln(x+1) transformed to satisfy homogeneity of variances, Cochran’s Test *C* = 0.18. *Post hoc* Student-Newman-Keuls (SNK) tests were done for significant sources of variation to examine the direction of differences relevant to hypotheses of interest (highlighted in bold).

x Denotes *post hoc* pooling when *P*>0.25. New *F* ratios are given for those tested against the pooled terms.

**Figure 4:**
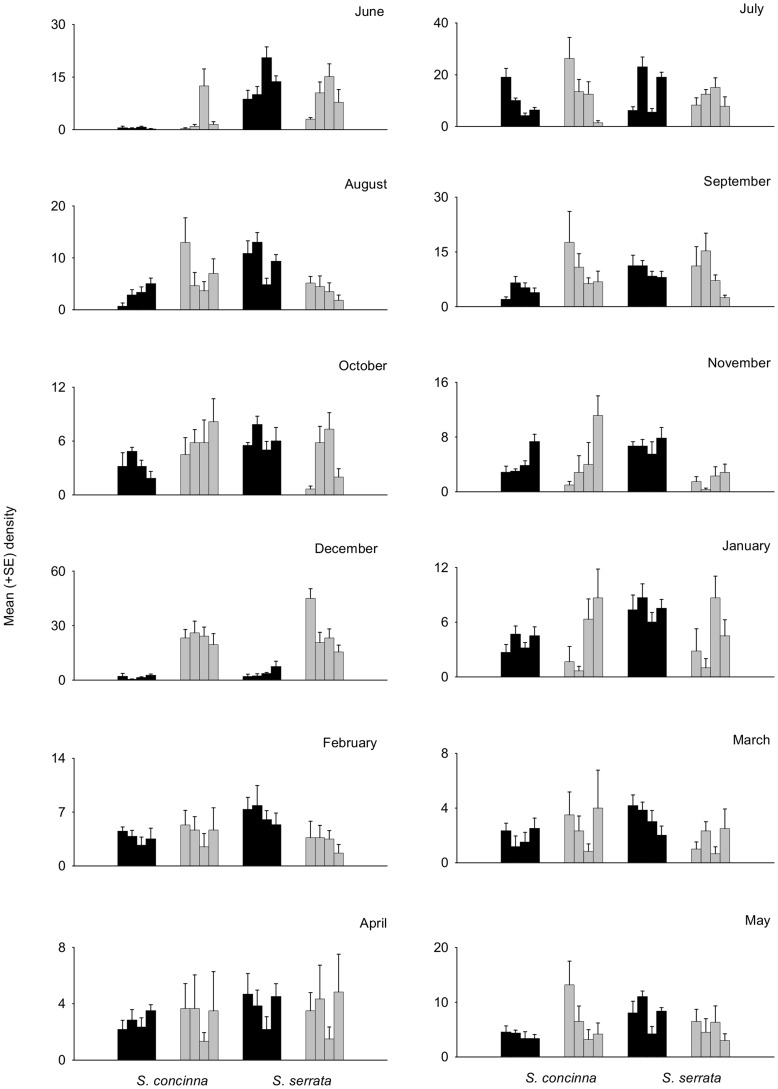
Mean (+SE; *n* = 6) density of adult *Siphonaria concinna* and *S. concinna* in small (black bars) or large patches (grey bars) in the Eastern Cape each month from June 2009 to May 2010.

**Figure 5:**
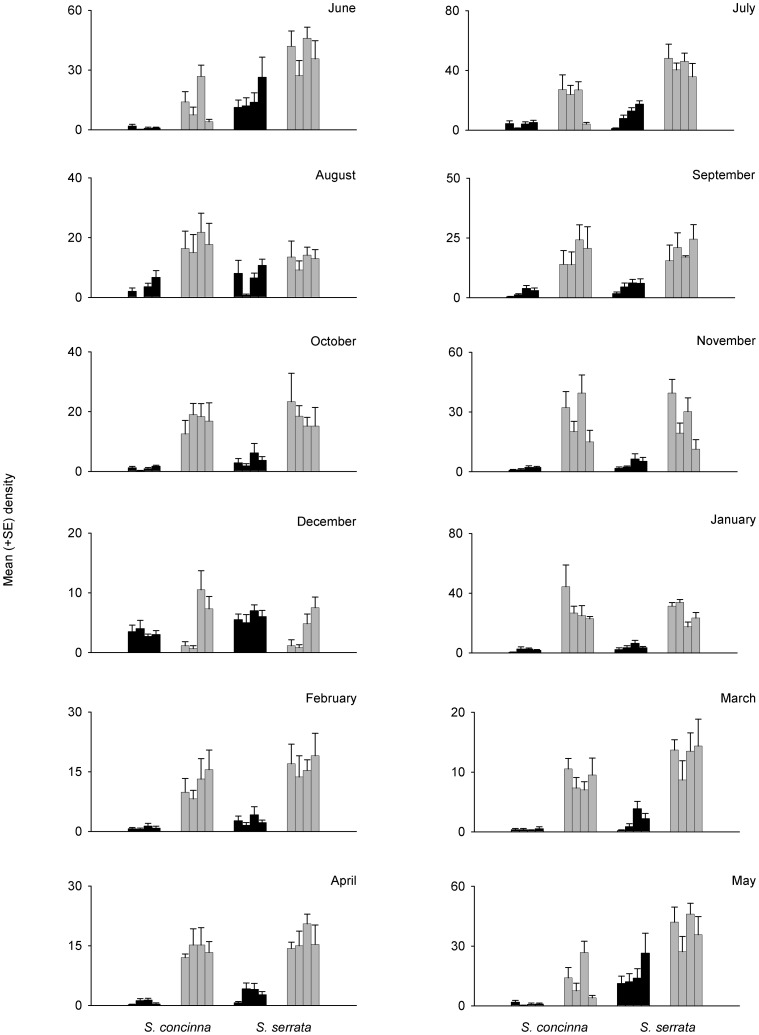
Mean (+SE; *n* = 6) density of juvenile *Siphonaria concinna* and *S. concinna* in small (black bars) or large patches (grey bars) in the Eastern Cape each month from June 2009 to May 2010.

In the Eastern Cape, densities of adult limpets did not differ between small and large patches at fixed monthly intervals throughout the year (Table 4, Fig. 4). There was a significant interaction between the factors Month and Patch, Month and Species, and also Species and Size (Table 4). With respect to the significant interaction between Species and Size, there were no differences in the density of *S. concinna* between small and large patches (Fig. 4, Table 4). There were, however, significantly greater densities of *S. serrata* in small patches than large (Fig. 4, Table 4). For juveniles, there was an interaction among the factors, Species, Month and Patch, and also Species by Month and Size (Table 5). In all months for both species, there were greater densities of limpets in large patches than small (Table 5), but clearly the magnitude of difference between small and large patches differed among months and between species (Fig. 5). Similarly, there was a Species by Month by Size interaction in the variance within patches (Table 6, Fig. 5). For both species and all months, limpet densities were generally more variable in large patches than small and variability was never higher in small patches (Table 6).

**Table 4:** Analysis of variance comparing densities of adult *Siphonaria concinna* and *S. serrata* over 12 months in the Eastern Cape region.

Source	d.f.	M.S.	*F*	*P*
Species = Sp	1	22.57	13.21	0.01
Month = Mo	11	12.15	5.51	0.00
Size = Si	1	17.56	9.33	0.02
Patch(Si) = Pa(Si)	6	1.88	1.88	0.08
Sp × Mo	11	3.67	3.67	0.00
Sp × Si	1	47.70	27.92	**0.00**
Sp × Pa(Si)	6	1.71	1.71	0.12
Mo × Si	11	0.86	0.39	0.96
Mo × Pa(Si)	66	2.21	2.21	0.00
Sp × Mo × Si	11	0.97	0.97	0.47
x Sp × Mo × Pa(Si)	66	1.04		
x Residual	960	1.00		
Total	1151			
Pooled	1026	1.00		
**SNK:** Species × Size				
*S. concinna*: Small = Large				
*S. serrata*: Small>Large				

Month was a fixed factor, Species (*S. concinna* or *S. serrata*) was fixed and orthogonal, Size (small or large) was fixed and orthogonal, and Patch (4 small and 4 large) was a random factor and nested in Region and Size, and *n* = 6 replicate quadrats. Data were Sqrt(x+1) transformed to satisfy homogeneity of variances, Cochran’s Test *C* = 0.04. *Post hoc* Student-Newman-Keuls (SNK) tests were done for significant sources of variation to examine the direction of differences relevant to hypotheses of interest (highlighted in bold).

x Denotes *post hoc* pooling when *P*>0.25. New *F* ratios are given for those tested against the pooled terms.

**Table 5:** Analysis of variance comparing densities of juvenile *Siphonaria concinna* and *S. serrata* over 12 months in the Eastern Cape region.

Source	d.f.	M.S.	*F*	*P*
Species = Sp	1	76.22	28.25	0.00
Month = Mo	11	13.39	5.82	0.00
Size = Si	1	1172.48	85.73	0.00
Patch(Si) = Pa(Si)	6	13.68	6.21	0.00
Sp × Mo	11	8.52	12.18	0.00
Sp × Si	1	3.98	1.48	0.27
Sp × Pa(Si)	6	2.70	1.22	0.29
Mo × Si	11	5.05	2.20	0.02
Mo × Pa(Si)	66	2.30	1.04	0.39
Sp × Mo × Si	11	1.82	2.60	**0.01**
Sp × Mo × Pa(Si)	66	0.70	0.32	0.00
Residual	960	2.20		
Total	1151			
**SNK**: Species x Month × Size				
All months and both species: Small<Large				

Month was a fixed factor, Species (*S. concinna* or *S. serrata*) was fixed and orthogonal, Size (small or large) was fixed and orthogonal, and Patch (4 small and 4 large) was a random factor and nested in Region and Size, and *n* = 6 replicate quadrats. Data were Sqrt(x+1) transformed to satisfy homogeneity of variances, Cochran’s Test *C* = 0.02. *Post hoc* Student-Newman-Keuls (SNK) tests were done for significant sources of variation to examine the direction of differences relevant to hypotheses of interest (highlighted in bold).

**Table 6:** Analysis of variance comparing within patch variability of *Siphonaria concinna* and *S. serrata* over 12 months in the Eastern Cape region.

Source	d.f.	M.S.	*F*	*P*
Species = Sp	1	0.33	0.51	0.48
Month = Mo	11	6.07	9.41	0.00
Size = Si	1	200.63	310.79	0.00
Sp × Mo	11	3.41	5.28	0.00
Sp × Si	1	2.43	3.76	0.05
Mo × Si	11	7.72	11.96	0.00
Sp × Mo × Si	11	2.70	4.18	**0.00**
Residual	144	0.65		
Total	191			
**SNK:** Species x Month × Size				
*S. concinna* – June–November and February–April: Small<Large				
December, January and May: Small = Large				
*S. serrata* - June, July and October–March: Small<Large				
August, September, April and May: Small = Large				

Month was a fixed factor, Species (*S. concinna* or *S. serrata*) was fixed and orthogonal, Size (small or large) was fixed and orthogonal, and *n* = 4 replicate patches. Data were Ln(x+1) transformed to satisfy homogeneity of variances, Cochran’s Test *C* = 0.12. *Post hoc* Student-Newman-Keuls (SNK) tests were done for significant sources of variation to examine the direction of differences relevant to hypotheses of interest (highlighted in bold).

## Discussion

The most striking pattern from this study, encompassing regional scales of 1500 km and temporal sampling for one year, is that two species with distinctly different modes of development and very different potential for dispersal generally showed the same responses to patch size. This suggests that siphonarian limpets respond to factors influencing their survival rather than processes influencing their arrival at patches. Alternatively, it is possible that different processes act on the two species to produce the same pattern independently.

In the regional analyses, differences between densities in large and small patches were apparent for both adults and juveniles across spatial scales but the pattern was stronger for adults than juveniles, presumably because adults and juveniles are influenced by different factors. Juveniles will be affected by the ease with which they can colonise patches, but adult densities will reflect both recruitment rates and post-recruitment effects, particularly competition. Thus, we expected large patches to have greater densities of *S. concinna* as they are easier for planktonic larvae to encounter given that they have largely passive dispersal, relying on currents and wind to move [28]. With respect to *S. serrata*, fecundity may also be greater in large patches. In an extensive review of the effects of habitat fragmentation by Fahrig [2], reproductive rates were generally greater in large patches than small patches and this was considered to be due to an increase in the amount of available habitat. Quinn [29] found the main cause of mortality of siphonarians was starvation, but the absence of clear effects of patch size on adult densities suggests that food does not limit these populations, nor that food availability on large patches is sufficient to support the greater densities of limpets. Other possible explanations include inter-specific competition with other intertidal grazers that out-compete *Siphonaria* spp. for food [22], or competition for space by sessile taxa [30]. Within patches, the densities of both species are generally quite similar, and therefore does not suggest that one species of *Siphonaria* is out-competing the other.

In addition to populations being denser in larger patches, they were also more variable within large patches. Our measurements were taken during low tides and these limpets show homing behaviour [31]. As heterogeneity of the rocky substrata may increase with increasing patch size, the availability of home scars may also be greater in large patches. Variable distributions in large patches also suggest an uneven distribution of resources, predators and/or recruitment. We would expect that large patches contain heterogeneous environments in which wave-action, algal food-supply and presence of predators vary at scales of centimetres to metres [32]. It is, however, unlikely that predation is the main cause of mortality of *S. serrata* and *S. concinna* in our study because *Siphonaria* spp. have chemical defences that render them unpalatable to predators [33]. Other factors that may also vary within patches may include pre-emption of space by sessile taxa [30] or non-biological factors, such as hydrodynamics and wave-exposure [34]. We would, however, need to invoke an effect of patch size on these factors in order to explain differences in limpet densities.

Based on a meta-analysis of terrestrial animals, Bender et al. [35] found that, for edge dwelling species, there was a strong relationship between patch size and population density. We would therefore expect that the effects of patch size might manifest more strongly towards the limits of a species’ geographic range, where it would seek to optimise habitat quality [36]. This did not appear to be the case for adult limpets, with no significant differences between small and large patches at either end of their distributions. For juveniles, there were greater densities in large patches than in small in the Western Cape (western limit) and Kwa-Zulu Natal (eastern limit), but this pattern also occurred in the centre of their distribution (Eastern Cape). Other regions, in between, showed either no difference or the opposite pattern. Conversely, this pattern occurred for juveniles in the middle of the geographic distribution, in the region where there were few limpets.

We expected that the two species of *Siphonaria* with different modes of development would respond differently to patch size. Across the entire range of the two species, they showed similar patterns of difference between small and large patches. When investigating temporal patterns in the Eastern Cape region, juveniles of the two species also showed similar responses. Adults throughout the year in the Eastern Cape did, however, show distinctly different responses to patch size. The broadcast spawner, *S. concinna* had similar densities in small and large patches. *S. serrata*, the brooder, had greater densities in small patches than large. These findings are also contrary to our predictions. We not only predicted differences between species but that *S. concinna* would show the greatest difference, with greater densities in large patches than in small. This is because as a broadcast spawner, it would be more likely to encounter large patches as pelagic larvae [37]. It is not clear why there were greater densities of adult *S. serrata* in small patches because as previously discussed fecundity should be greater in large patches.

Patterns were clearer for monthly sampling within the Eastern Cape region than across spatially separated regions. This suggests that sites may show the same patterns consistently through time and that temporal generality is possible. Generalisations to other regions within the distribution range of the two species of *Siphonaria*, is however, more difficult. Apart from possible differences in the factors driving patterns of patch use at these small, within-region scales, regions also differ with respect to large scale processes, e.g. biogeographic trends in temperature, climate, past-history and oceanographic conditions [32]. For example, the areas which were sampled in the Eastern Cape and the South-western Cape are influenced by upwelling [38], whereas the two Transkei regions have low productivity [39]. Some such factors may well interact with the effects of patch size.

In conclusion, the effects of patch size on densities and variability of populations of limpets were similar regardless of the mode of development. This is contrary to our predictions and implies that the ability to disperse need not have an overriding influence on habitat use and consequent densities among patches of a larger metapopulation. In many parts of the world, large scale movement of sand has very powerful effects on rocky shore communities, including altering patch sizes through burial of rocks, and can be driven by modification of coastal habitats by the introduction of groynes, breakwaters and piers [40], [41], [42]. We suggest the importance of understanding patterns of distribution of species with respect to habitat heterogeneity in linear landscapes. In linear landscapes such as coastlines, the ways in which changes to habitat availability and habitat patch size affect species may be more generalised and depend less on species mobility than expected.
